# Characterization of Children’s Exposure to Extremely Low Frequency Magnetic Fields by Stochastic Modeling

**DOI:** 10.3390/ijerph15091963

**Published:** 2018-09-08

**Authors:** Marta Bonato, Marta Parazzini, Emma Chiaramello, Serena Fiocchi, Laurent Le Brusquet, Isabelle Magne, Martine Souques, Martin Röösli, Paolo Ravazzani

**Affiliations:** 1 Istituto di Elettronica e di Ingegneria dell’Informazione e delle Telecomunicazioni IEIIT CNR, 20133 Milano, Italy; marta.parazzini@ieiit.cnr.it (M.P.); emma.chiaramello@ieiit.cnr.it (E.C.); serena.fiocchi@ieiit.cnr.it (S.F.); paolo.ravazzani@ieiit.cnr.it (P.R.); 2 Dipartimento di Elettronica, Informazione e Bioingegneria DEIB, Politecnico di Milano, 20133 Milano, Italy; 3 Laboratoire des Signaux et Systèmes (L2S), CentraleSupélec, CNRS, Univ. Paris-Sud, Université Paris-Saclay, 91192 Gif-sur-Yvette, France; laurent.lebrusquet@centralesupelec.fr; 4 Medical Studies Department of EDF (Electricite de France), 92300 Levallois-Perret, France; isabelle.magne@edf.fr (I.M.); martine.souques@edf.fr (M.S.); 5 Department of Epidemiology and Public Health, Swiss Tropical and Public Health Institute, 4051 Basel, Switzerland; martin.roosli@swisstph.ch; 6 University of Basel, 4001 Basel, Switzerland

**Keywords:** children’s exposure, ELF-MF, stochastic model, kernel density estimation, *p*-value histogram

## Abstract

In this study, children’s exposure to extremely low frequency magnetic fields (ELF-MF, 40–800 Hz) is investigated. The interest in this thematic has grown due to a possible correlation between the increased risk of childhood leukemia and a daily average exposure above 0.4 µT, although the causal relationship is still uncertain. The aim of this paper was to present a new method of characterizing the children’s exposure to ELF-MF starting from personal measurements using a stochastic approach based on segmentation (and to apply it to the personal measurements themselves) of two previous projects: the ARIMMORA project and the EXPERS project. The stochastic model consisted in (i) splitting the 24 h recordings into stationary events and (ii) characterizing each event with four parameters that are easily interpretable: the duration of the event, the mean value, the dispersion of the magnetic field over the event, and a final parameter characterizing the variation speed. Afterward, the data from the two databases were divided in subgroups based on a characteristic (i.e., children’s age, number of inhabitants in the area, etc.). For every subgroup, the kernel density estimation (KDE) of each parameter was calculated and the *p*-value histogram of the parameters together was obtained, in order to compare the subgroups and to extract information about the children’s exposure. In conclusion, this new stochastic approach allows for the identification of the parameters that most affect the level of children’s exposure.

## 1. Introduction

Children’s exposure to extremely low frequency magnetic fields (ELF-MF, 40–800 Hz) has been a topic of high interest since the late 1970s, when ELF-MF (in particular from power lines sources) was studied as a possible health risk factor for childhood leukemia [1]. Leukemia is the most common type of childhood cancer, accounting for 30% of all cancers diagnosed in children younger than 15 years old, but still little is known about its etiology. Several meta-analyses found that there is an increased risk of childhood leukemia’s onset for daily average exposure above 0.4 µT, without any causal relationship [2,3,4]. Indeed, the reason of the correlation between the ELF-MF exposure and the increased risk is still uncertain [5]. Based on these results, in 2002 the International Agency for Research on Cancer (IARC) concluded that ELF-MF should be classified as “possibly carcinogenic to humans” based on “limited evidence of carcinogenicity in humans” and “inadequate evidence of carcinogenicity in experimental animals” [6]. This evaluation has recently been confirmed by the EC FP7 Project ARIMMORA [7]. In this context and to better understand the possible association of the magnetic field (MF) exposure and childhood leukemia, different studies were performed to provide data about children’s personal exposure in Europe [8,9], North America [10,11,12,13,14,15], and Asia [16,17,18]. In the studies, the level of the magnetic field to which the children were exposed was estimated. The magnetic field at extremely low frequency is indeed the typical metric used for environmental exposure, as reported in all current international guidelines for maximum exposure level for human exposure (see, e.g., the ICNIRP guidelines [19]). Other previous studies consisted of measurements performed only in the child’s bedroom [20,21]. More recently, two new measurement campaigns were carried out in Europe to investigate the level of children’s exposure to ELF-MF in their daily life, caused by mainly the presence of power lines [22,23] or by all sorts of magnetic field sources [24], measuring magnetic field amplitudes through personal dosimeters worn by the children themselves. In the first one, conducted in the framework of the FP7 European Project ARIMMORA, a personal measurement campaign of children’s exposure was implemented in Italy and Switzerland [22,23]. Data collected revealed that the average geometric mean for children’s personal ELF-MF exposure was 0.04 µT and exposure in bedrooms was 0.05 µT. The second one was conducted in France in the framework of the project EXPERS (EXPosition PERSonelle in French). Data collected revealed that the arithmetic mean and the geometric mean for children’s personal ELF-MF exposure were 0.09 µT and 0.02 µT, respectively [24].

The aim of this study was to develop a new method to characterize children’s exposure to ELF-MF starting from personal measurements by stochastic modeling based on segmentation and to apply it to the two personal measurements of children’s ELF-MF exposure mentioned above (projects ARIMMORA and EXPERS).

The developed stochastic model was fundamental in advanced characterization of children’s ELF-MF exposure, facilitating a comparison between different exposure conditions (e.g., day vs. night, different towns, and living areas), providing also the ability to better explain differences between subgroups of children to individuate the most discriminant ones. Last but not least, the stochastic approach allowed for the generation of models of children’s exposure based on data from few specific measurement campaigns, providing generalized information about the exposure of children of similar groups/environment/locations.

## 2. Materials and Methods

### 2.1. Data Sources

In the present study, two databases of measurements of children’s personal exposure to extremely low frequency magnetic fields are considered. The first database is the one coming from the project ARIMMORA (in the following called ARIMMORA database) [22,23], which gathered 48 h or 72 h recordings of children’s personal exposure to ELF-MF in Milan (Italy) and in Basel (Switzerland). The children’s age was between 5 and 14 years old, for a total of 166 children. The recordings were collected both in winter and summer seasons from 1 April 2012 and 20 December 2013. The second database is the one coming from the project EXPERS (in the following called EXPERS database) [24], which is composed of French children aged between 0 and 14 years old, located throughout the country and recorded over 24 h. The EXPERS database collected the registrations of 948 children in different locations of France, located in rural places and in urban places, and of 29 children in Paris, for a total of 977 children. The measurements were performed during cold seasons (February–April 2007, October 2007–April 2008, and October 2008–January 2009). Table 1 summarizes some characteristics of the two databases.

In both measurement campaigns, the children wore the same device, an EMDEX II (Enertech, Campbell, CA, USA), that was set to measure broadband (40–800 Hz) and harmonic (100–800 Hz) magnetic field amplitudes and had a sensitivity range from 0.01 to 300 µT. In the EXPERS project, the children wore the device over 24 h, whereas in the ARIMMORA project, the measurement time was of two or three days. The sample rate was set to 3 s for the EXPERS database and to 30 s for the ARIMMORA database. For both databases, additional information were collected; in particular, a questionnaire about the subject and his/her home’s characteristics (such as children’s age, number of people in the house, type of home, year of home construction, living place, and distance from MV/LV (20 kV/400 V) substation) was compiled and the subject (or the parents) filled in a timetable with details about daily activities, with the duration and the location. Some of this additional information has been used in the following to identify subgroups of data with specific features.

### 2.2. Data Processing

**Figure 1:**
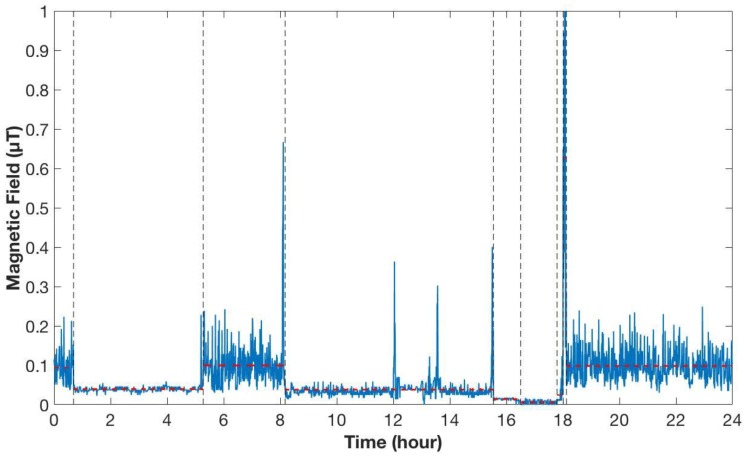
An example of 24 h children’s personal ELF-MF exposure measurement from the ARIMMORA database. The vertical line in black represent the point of change in the signal (called change-points) and the dotted red line represents the segments obtained from the signals.

From each recording, only full days of personal measurements were extracted (from 00:00 to 24:00), which means 2880 values for each recording for the ARIMMORA database (since the sample rate was 30 s) and 28,800 values for each recording for the EXPERS database (since the sample rate was 3 s). Moreover, registrations that were too short (less than 24 h) were eliminated, and registrations that were too long were cut at midnight. Furthermore, for the EXPERS database, the registrations where the alarm clock was too close to the children’s bed (less than 50 cm away) were also excluded, as in the protocol of the study of Magne et al. (2017) [24]. The complete days of registrations from the two databases resulted in 682 days for the ARIMMORA database and 767 days for the EXPERS database, for a total of 1449 full days. In Figure 1, an example of 24 h personal measurement from the ARIMMORA database is shown.

From the example in Figure 1, it can be seen that the personal measurement of children’s exposure to ELF-MF were typically characterized by abrupt changes in the mean level and in the dispersion around the mean. The approach here used to characterize the level of magnetic field in real exposure scenarios consisted, therefore, in detecting these change points and modeling every segment individuated between two change-points with an autoregressive (AR) model. The parameters of each AR model obtained were divided in different subgroups in order to evaluate the differences and the similarities between them with both qualitative and quantitative techniques. The following steps of signal processing were performed:

1. change-point detection and modeling of each segment with an AR model;
2. calculation of kernel density estimation (KDE) of the AR model parameters (qualitative analysis);
3. calculation of *p*-value histograms of the parameters obtained from the AR model (quantitative analysis).

This process was repeated for every subgroup of data that was analyzed. In the following paragraphs, every step will be discussed in detail.

#### 2.2.1. Change-Point Detection and AR Modeling

The change-points are the time points that divided the measurements of the databases into distinct homogeneous segments (or equivalent stationary segments) [25]. Change-point detection has been deeply investigated in the literature since it can be used in many applications, including financial data [26], climate data [27], biomedical data [28], and signal processing [29]. There are different approaches to detect change-points (for a survey see Basseville et al., 1993 [30]), such as Binary Segmentation [31], Circular Binary Segmentation [32], Wild Binary Segmentation [26], and the Pruned Exact Linear Time method (PELT) [27,33,34]. In this work, we used the PELT procedure, because of its ability to jointly estimate the number of change-points, their locations, and the AR model parameter of each segment. The PELT approach here used was based on the algorithm of Jackson and colleagues [34]. It can be applied to find change-points under a range of statistical criteria such as penalized likelihood, quasi-likelihood, and the cumulative sum of squares. This algorithm effectively captures the change in mean, variance, and mean-variance.

In summary, the segmentation process aims to

- find the periods of stability and homogeneity in the behavior of the time series;
- identify the change-points;
- represent the regularities and features of each segments (estimate the model of each segment by parameters like change-points location, segment mean and variance, and segment duration for each day of recording).

Each recording from the two databases was ordered in a sequence way, as $y\;=\;\left(y_{1},\ldots ,y_{n}\right)$. The following notation was used: for *s* ≥ *t*, the set of observations from time *t* to time *s* is: $y_{t:s}\;=\;\left(y_{t},\ldots ,y_{s}\right)$. If it is assumed that there were *k* change-points in the data, the data were split into *k* + 1 distinct segments. Let $\tau_{j}$ be the location of the $j^{th}$ change-point for *j* = *1*,…,*k.* With previous notation, this $j^{th}$ segment represented the data points $y_{\tau_{j-1}+1:\tau_{j}}\;=\;\left(y_{\tau_{j-1}+1},\;\ldots ,\;y_{\tau_{j}}\right)$. The setting was $\tau_{0}=0$ ($\tau_{0}$ + 1 is thus the first point of the data vector) and $\tau_{k\;+\;1}$ = *n* (last point of the data vector). Let $\tau =\left(\tau_{0},\ldots ,\tau_{k+1}\right)$ be the set of change-points to be estimated. A generic $y_{t}$ can be defined by the sum of *k* segments (each of which modeled by an AR model of order *q* for the $j^{th}$ segment):

(1) $$y_{t}\;=\;\{\begin{array}{c} \mu_{1}\;+\;\overset{q_{1}}{\underset{r\;=\;1}{\sum }}\varphi_{1r}\left(y_{t\;-\;r}\;-\;\mu_{1}\right)\;+\;\sigma_{1}z_{t},\;t\in \left[\tau_{0},\tau_{1}\right]\\ \mu_{k}\;+\;\overset{q_{k}}{\underset{r\;=\;1}{\sum }}\varphi_{kr}\left(y_{t\;-\;r}\;-\;\mu_{k}\right)\;+\;\sigma_{k}z_{t},\;t\in [\tau_{k\;-\;1},\tau_{k}] \end{array}$$

where *k* is the number of segments, $\tau_{j}$ is the location of the $j^{th}$ change point, $q_{j}$ is the order of the AR model, $\mu_{j}$ is the mean of the stationary process, $\varphi_{j}$ is the coefficient of the AR model, *σ*^2^ is the noise variance for each $j^{th}$ segment, and $z_{t}$ is the noise sequence, independent and identically distributed, with zero-mean and unit-variance. In our case, the AR model was chosen of the first order, so $q_{j}$ = 1 ∀*j*.

The number of change-points detected through the PELT algorithm implemented in R was of 52,469 points for the whole dataset. It means that the change-points process allows for an exhibition of about 52,470 segments (events). After this process, every segment was described by these four parameters:

1. the mean of the stationary process (μ);
2. the variance of the stationary process (*σ*^2^);
3. the coefficient of the AR model of order 1 (ϕ);
4. the duration of the stationary process (T).

Every segment was so identified by *p* = (μ, *σ*^2^, φ, T) and the whole dataset included ($p_{1}$, …, $p_{52470}$). The choice of these four parameters for describing the obtained segments was related to the use of the piecewise autoregressive model. The parameters necessary to describe an AR model are indeed the duration of the segment and the coefficients of the AR model, which are the amplitude or mean, noise variance, and the autoregressive coefficients, which in this case are only one, because the AR model is of order 1 [25].

#### 2.2.2. Calculation of Kernel Density Estimations of the Four Parameters

The kernel density estimation (KDE) of the 4 parameters of each segment was estimated. The kernel density estimation is a nonparametric technique for probability density function estimation [35,36]. In detail, let $X_{1},X_{2},\ldots ,X_{n}$ in the set R be a univariate random sample from a distribution with density *f*, which we wish to estimate. The kernel density estimation $\hat{f}$: R → R is given by

(2) $$\hat{f}\left(x\right)\;=\;\frac{1}{n}\overset{n}{\underset{i\;=\;1}{\sum }}K_{h}\left(x\;-\;X_{i}\right)\;=\;\frac{1}{nh}\overset{n}{\underset{i\;=\;1}{\sum }}K\left(\frac{\left(x\;-\;X_{i}\right)}{h}\right)$$

where *n* is the number of samples, *h* is the bandwidth or smoothing parameter of the kernel function, and *K* is the kernel function that satisfies the following condition:

(3) $$\int_{-\infty }^{+\infty }K\left(x\right)dx\;=\;1.$$

The types of kernel functions and the bandwidth *h* are important factors in determining the accuracy of the estimated function. In fact, if *h* becomes small, the kernel function becomes sharp. If *h* becomes large, the kernel function becomes smooth, so an inappropriate bandwidth yields under-smoothing or over-smoothing, so an optimum bandwidth must be determined. In the multivariate case ($X_{i}\;=\;\left(X_{i,1},\ldots ,X_{i,d}\right)\in R^{d})$, a common choice is to consider a multivariate kernel $K_{d}\left(x\right)$ as a product of univariate kernels: $K_{d}\left(x\right)\;=\;\overset{d}{\underset{j\;=\;1}{\prod }}K\left(\frac{x_{j}}{h_{j}}\right)$.

In our case, we used a Gaussian kernel for the function K(.) and $h_{j}$ was calculated from the empirical rule given in [37]:

(4) $$h_{j}=\sigma_{j}\cdot n^{\frac{-1}{\left(d+4\right)}}$$

where *σ_j_* is the standard deviation of the samples for the $j^{th}$ variable, and *d* is the number of parameters, which in our case was set to 4.

#### 2.2.3. Calculation of *p*-Value Histograms of the 4 Parameters

In order to test if two different subgroups have the same characteristics and can be modeled with the same model, it was necessary to check if two multivariate samples were drawn from the same distribution. In particular, in this study, we used the test presented by Baringhaus and colleagues [38], which allows for a comparison of 2 samples of $R^{d}$. Let X a $n_{x}\;\times \;d$ matrix for the first sample and Y a $n_{y}\;\times \;d$ matrix for the second one. The output is a *p*-value, which can be used to decide if the null hypothesis (i.e., the two sample are taken from the same distribution) is accepted or not.

In the present work, the described procedure was applied on the vector of the four parameters. In order to determine that the lengths of the samples do not affect the results and to reduce the processing time, a Monte-Carlo procedure was also applied. Specifically, 200 events from each sample of the considered subgroups were randomly picked before applying the test. This procedure was repeated 3000 times to obtain the *p*-value histogram. If the null hypothesis is true, then the *p*-value histogram is expected to be approximately uniform on the interval from 0 and 1, whereas if there is a clustering of *p*-value near zero, the null hypothesis is false [39]. Moreover, for the uncertain cases examined, the false discovery rate (FDR) was calculated [40]. The FDR is the expected value of the proportion of false positive features among all those called significant. By significant features we mean the ones where the null hypothesis is rejected in favor of the alternative hypothesis.

### 2.3. Data Analysis

The datasets of children’s exposure to ELF-MF was divided in different subgroups of segments with specific characteristics. The different subgroups analyzed and the number of segments for each subgroup are described in Table 2.

As can seen from Table 2, the comparisons made here are between the following:

- Segments regarding daytime (from 07:00 to 21:00 h) and segments regarding night time recordings (from 21:01 to 06:59 h) considering the whole database.
- Segments divided by the children’s age from whole database. Three groups were analyzed: children from 0 to 4 (these data were only from the EXPERS database), children from 5 to 9, and children from 10 to 14.
- Segments divided by the number of inhabitants of the town where the children were living. In this case, the ARIMMORA database was split into segments regarding the measurements in Milan and the ones in Basel. The EXPERS database was split into segments regarding the measurements in Paris and in the rural area (less than 2000 inhabitants), and into different groups based on the number of town’s inhabitants, as shown in Table 2.
- Segments divided by the distance between the children’s domicile and the nearest ML/LV substation. As can seen from Table 2, for this last analysis, only the EXPERS database was considered. The EXPERS database was split into three groups. The first group collected the segments obtained from recordings of children whose domicile was at least 40 m away from the substation; the second group were the segments obtained from the children’s recordings where the substation is less than 40 m away from the children’s domicile; the last group were the segments obtained from the children’s recordings where the substation is in the same building of children’s domicile or adjacent.

## 3. Results and Discussion

### 3.1. Daytime Versus Nighttime

**Figure 2:**
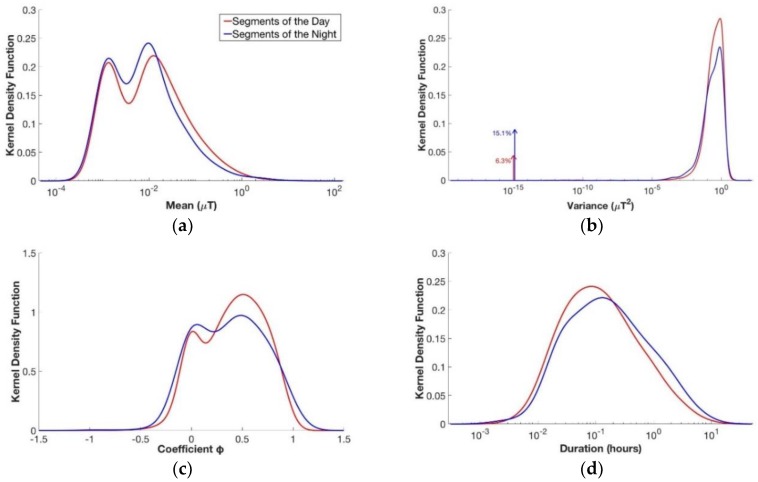
Estimated kernel density function for the four variables of the segments of the day (in red) compared with estimated kernel density function for the four variables of the segments of the night (in blue); from above to down, left to right: (**a**) mean µ, (**b**) variance *σ*^2^, (**c**) coefficient φ, and (**d**) duration T.

As shown in Table 2, here we evaluated the differences or similarities between the segments regarding the daytime and night time measurements from the whole database of children personal exposure measurements. The estimated kernel density of the four parameters (i.e., mean μ, variance *σ*^2^, coefficient φ, and duration T) are shown in Figure 2. They correspond to the full KDE density projected to each of the four parameters.

**Figure 3:**
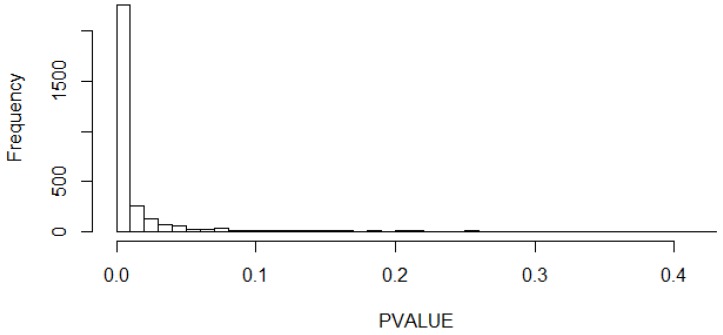
*p*-value histogram for the comparison between the daily and nightly segments.

As can be seen from the figure, the curves are characterized by the presence of peaks, which indicated the values with the highest probability of occurrence for each parameter. In particular, it can be seen that the mean value μ of the segments’ level of magnetic field presented slightly higher values during the day than during the night. In fact, the second peak of the kernel density function of the mean for the daily segments had a value around 0.013 µT, whereas for the nightly segments it was at about 0.010 µT. The mean level for the day segments’ KDE is around 0.070 µT, whereas for the night segments’ KDE is around 0.053 µT. For both functions, values higher than 0.4 µT, which is the value above which there is an increased risk of onset of childhood leukemia, were quite unlikely. The kernel density functions of the variance (*σ*^2^) presented the peaks almost in the same position for the two different subgroups of segments. In fact, the peak had a value of 0.60 μT^2^ for the nightly segments and 0.78 μT^2^ for the daily ones. The higher variance value for the daily segments could be explain since during the day time there is a presence of more activities than during the night, so there was more variability. Furthermore, in the kernel density functions of the variance (*σ*^2^), the presence of two Dirac pulses can be seen, one for the day group and one for the night group. It was decided to use this representation to indicate the percentage of the variance data, which had a value under the limit of resolution of calculation. This choice was necessary to avoid a false representation, which would lead to a misunderstanding of the results. These data represented a really low variation of the variance parameters (the order is around 10^−15^), so they are not so significant for estimating the level of magnetic field to which children are exposed. The impulses indicated a higher variability at low values for the segments of the night compared with the segments of the day. The kernel density function for the coefficient φ, representing the segments’ variation speed, were quite different between day and night. Moreover, it can be seen that the curves of coefficient φ exceeded the value of 1, although this indicated an inadequate modeling of the segments. This is due to the way of calculation of kernel density estimation and not for a real presence of coefficients φ with a value higher than 1, which were eliminated during the preliminary phase of modeling the segments. The duration parameter *T* showed higher values for the segments of the night, compared to the ones of the day. This can be explained by the fact that, during the night, there is less activity and less variability, so the characteristics of the segments remain the same for longer durations. This qualitative analysis was also confirmed by the statistical evaluation. In fact, the *p*-value histogram obtained from the comparison between the daytime and night time groups, considering the four parameters all together, showed a clustering of *p*-values near zero (see Figure 3), which means that there is a difference between the daily and nightly segments. This was also confirmed from the calculation of FDR, which had a value of 4.5 × 10^−5^. For this reason, in the next analyses, the daily and nightly segments were always considered separately.

### 3.2. Children’s Age

**Figure 4:**
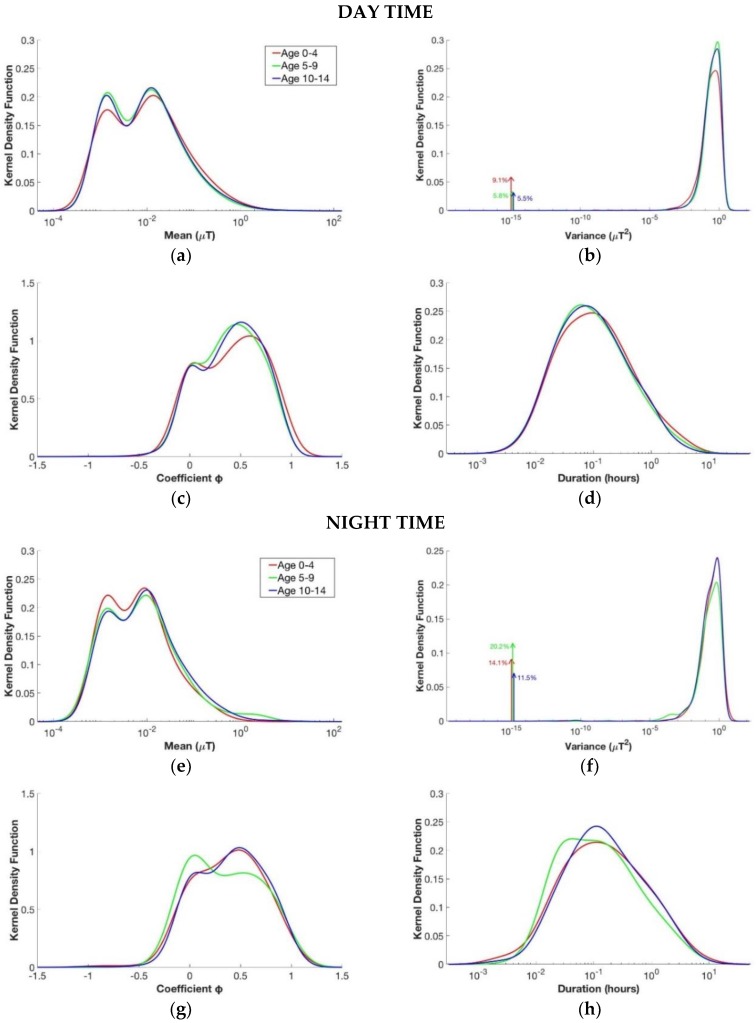
Estimated kernel density function for the four variables of the segments of the day in the upper part and for the segments of the night in the lower part. From above to down, left to right: (**a**,**e**) mean µ, (**b**,**f**) variance *σ*^2^, (**c**,**g**) coefficient φ, and (**d**,**h**) duration T. The groups considered are children with age between 0 and 4 years old (in red), children with age between 5 and 9 years old (in green), and children with age between 10 and 14 years old (in blue).

This analysis compared the measurement segments divided according to children’s age (as reported in Table 2) from the whole database of children personal exposure measurements. Figure 4 shown the kernel density estimation for each age group considering separately daily and nightly segments.

Based on Figure 4, it can be concluded that the kernel density function of the mean of the segments’ level of the magnetic field for the three groups both for daily and nightly segments were quite similar. During the day, the second peak of the three curves was always around 0.013 µT. During the night, the second peak of the functions was slightly lower than that during the day, and it was around 0.010 µT for children between 5 and 9 years old and for those between 10 and 14 years old and 0.009 µT for children between 0 and 4 years old. For the other parameters, there were not so many differences, except for the curve of the φ coefficient of nightly segments for children between 5 and 9 years old and daily segments for those between 0 and 4 years old. Although from the qualitative analysis it seemed that the three groups had quite similar characteristics, the analysis of the *p*-value histogram of the four parameters considered together proved that the three groups were not similar. In fact, the *p*-value histograms obtained from the comparison between the three age groups both for daily and nightly segments were skewed toward zero in all comparisons. Children between 0 and 4 years old, in particular, were more different from the other two groups of age.

### 3.3. Number of Inhabitants

**Figure 5:**
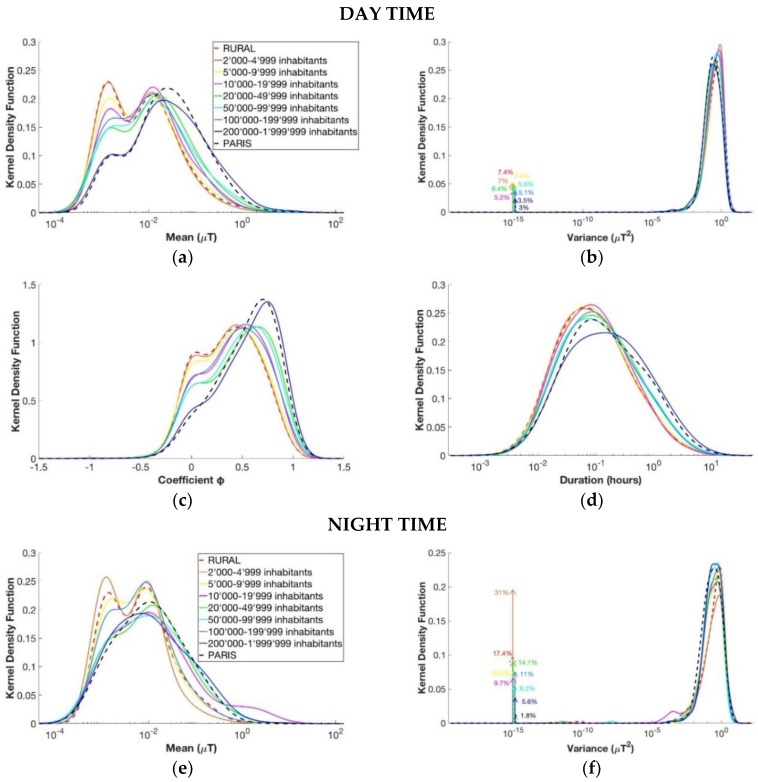

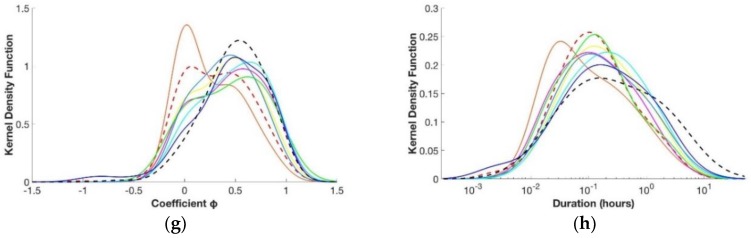
Estimated kernel density function of the four variables for the segments of the day in the upper part and for the segments of the night in the lower part. From above to down, left to right: (**a**,**e**) mean µ, (**b**,**f**) variance *σ*^2^, (**c**,**g**) coefficient φ, and (**d**,**h**) duration T. The groups considered are divided according to the number of inhabitants of the town in which the children lived as shown in Table 2.

This analysis compared the measurement segments in function of the number of inhabitants of the town in which the children lived (see Table 2). Figure 5 reported the KDE for the four parameters for each subgroup, considering separately daily and nightly segments from the database EXPERS.

For the daily segments, it can be noted that, for the kernel density function of the mean, the higher the number of inhabitants of the town was, the higher the segments’ level of magnetic field was. Indeed, the second peak of these functions, which indicated the highest value of exposure with the highest probability, was of 0.012 µT for the rural area and about 0.026 µT for Paris, more than double the rural area. In general, the lower the number of inhabitants was, the more similar the curves were to those of the rural area, whereas the higher the number of inhabitants was, the more similar the curves became to the curve of Paris. This qualitative analysis was also confirmed from the *p*-value histogram analysis and the FDR calculation. In fact, for the daily segments, the rural area was not statistically different from the group of 2000–4999 inhabitants (with a *p*-value histogram approximately uniform and an FDR value of 0.86) and from the group of 5000–9999 inhabitants (with a *p*-value histogram approximately uniform and an FDR value of 0.43). Other comparisons of the daily segments that showed similarity between groups were the comparison between the groups of 2000–4999 inhabitants and of 5000–9999 inhabitants (with a *p*-value histogram approximately uniform and an FDR value of 0.44) and between the groups of 20,000–49,999 inhabitants and of 50,000–99,999 inhabitants (with a *p*-value histogram approximately uniform and an FDR value of 0.56), which means that these segments have the same characteristics. Based on these results, children living in towns with a number of inhabitants up to 9999 are all exposed to ELF-MF to the same extent. Similarly, children living in a town with a number of inhabitants between 20,000 and 99,999 are exposed to ELF-MF to a similar extent. Regarding comparison with the nightly segments, it can be seen that the means of the segments’ level of magnetic field were more similar among the different groups than they were during the day, but there was more variability in the kernel density function of the segments’ duration T. During the night, the *p*-value histogram analysis revealed that the subgroups identified by the number of inhabitants are statistically different among them.

**Figure 6:**
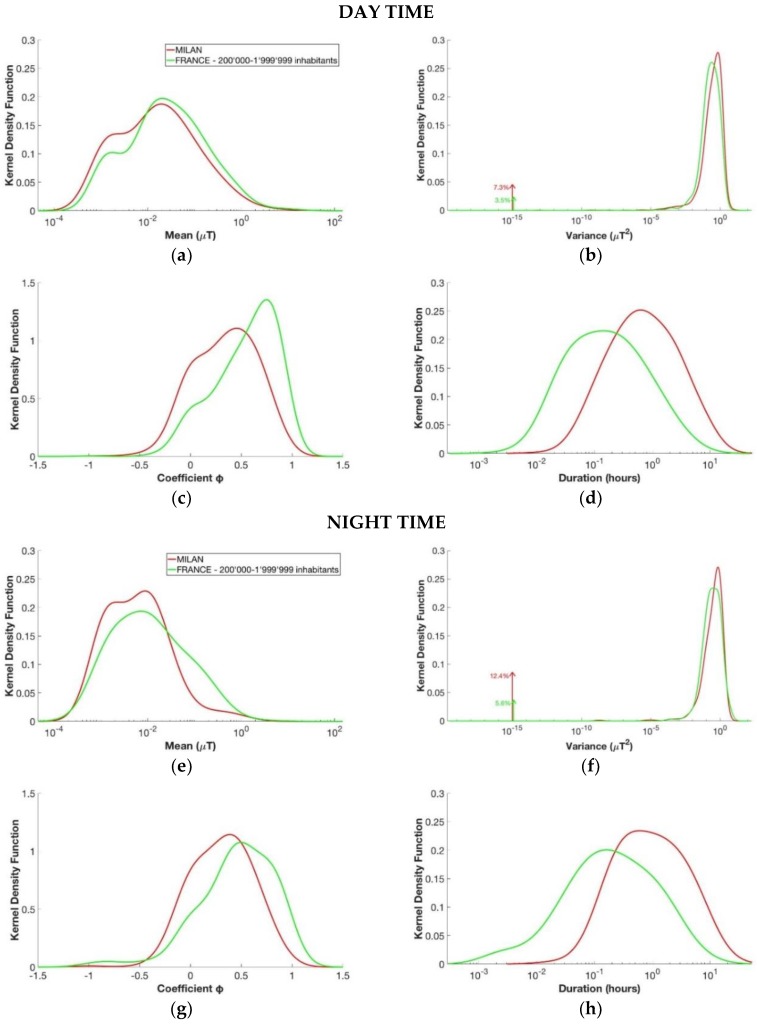
Estimated kernel density function for the four variables of the segments of the day in the upper part and for the segments of the night in the lower part. From above to down, left to right: (**a**,**e**) mean µ, (**b**,**f**) variance *σ*^2^, (**c**,**g**) coefficient φ, and (**d**,**h**) duration T. The groups considered are those with registration of Milan from the ARIMMORA database (in red) and the group of 200,000–1,999,999 inhabitants from the EXPERS database (in green).

Figure 6 illustrates the comparison between the KDE for the segments obtained from the recordings regarding the city of Milan and the corresponding group of number of inhabitants in France, which is the group with 200,000–1,999,999 inhabitants (the population of Milan is about 1,347,000 inhabitants). For the day, the mean of the segments’ level of magnetic field was similar between the two groups. In fact, the peak of the density kernel function of the mean was about 0.020 µT for Milan and of 0.021 µT for the corresponding group of inhabitants in France. During the night, the segments’ level of magnetic field in France was a bit higher than the one in Milan. The duration T of each segment is higher for Milan during both day and night. This could be due to the different sample rate of the two databases (3 s for the EXPERS database and 30 s for the ARIMMORA database). Indeed, with shorter time sampling, more changes in the mean level and in the dispersion around the mean were captured for the EXPERS database, which resulted in segments with shorter durations compared with the segments of the ARIMMORA database.

Although the segments were obtained from cities that belong to the same category by the number of inhabitants, the two groups were statistically different and cannot be modeled with the same model, since the *p*-value histograms were clustered near zero for both day and night segments.

**Figure 7:**
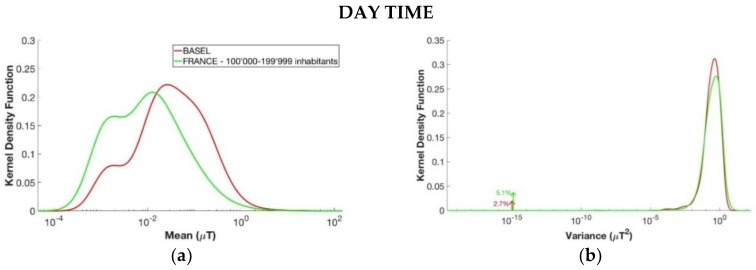

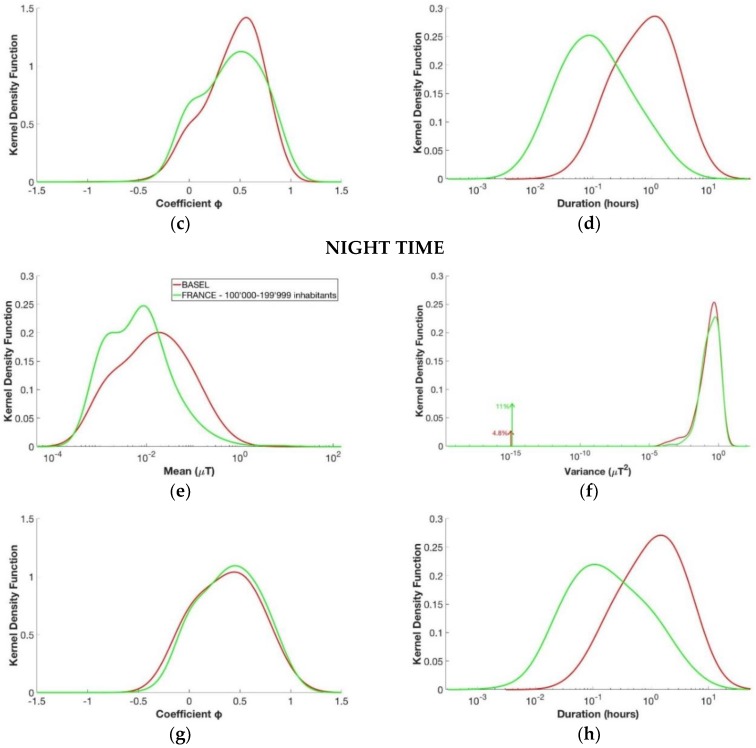
Estimated kernel density function for the four variables of the segments of the day in the upper part and for the segments of the night in the lower part. From above to down, left to right: (**a**,**e**) mean µ, (**b**,**f**) variance *σ*^2^, (**c**,**g**) coefficient φ, and (**d**,**h**) duration T. The groups considered are the those with registration of Basel from the ARIMMORA database (in red) and the group of 100,000–19,999 inhabitants from the EXPERS database (in green).

Similarly, a comparison between the data of Basel (the population in Basel is around 171,017 inhabitants) and the class of 100,000–199,999 inhabitants from the EXPERS database was conducted, and the results are shown in Figure 7. In this case, the mean of the segments’ level of magnetic field in Basel was higher than the one for the same category by the number of inhabitants in France, both for day and night. The peaks of the density kernel function of the mean were in fact 0.22 µT for the day and 0.019 µT for the night in Basel, whereas for the EXPERS data the values were 0.013 µT for the day and 0.009 µT for the night. The considerations on the kernel density function of the segments’ duration T were the same as those of the previous analysis about Milan and the corresponding class of inhabitants in France, with a longer duration for Basel. Furthermore, the *p*-value histograms were skewed toward zero also in this case, and the two groups were therefore statistically different.

### 3.4. Distance from ML/LV Substation

**Figure 8:**
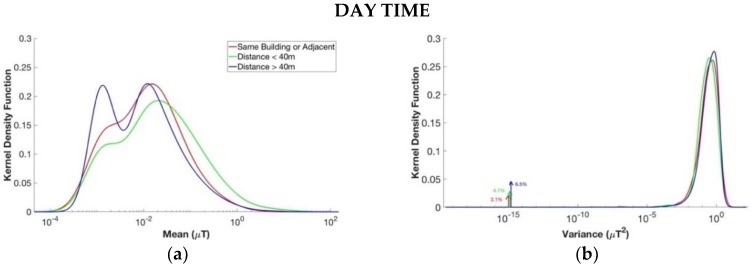

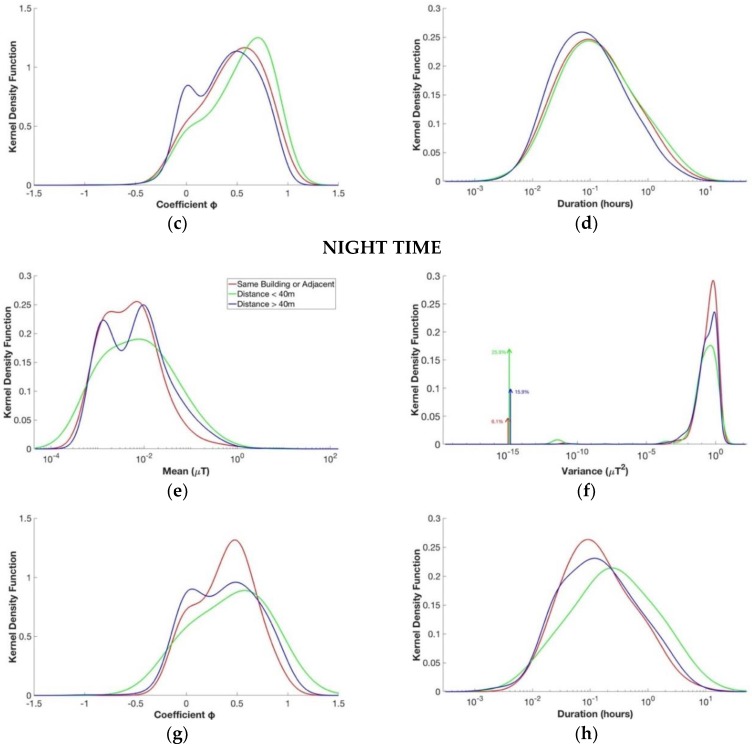
Estimated kernel density function for the four variables of the segments of the day in the upper part and for the segments of the night in the lower part. From above to down, left to right: (**a**,**e**) mean µ, (**b**,**f**) variance *σ*^2^, (**c**,**g**) coefficient φ, and (**d**,**h**) duration T. The groups considered are measurements where the children’s domicile was at least 40 m away from the substation (in blue), measurements where the substation was less than 40 m away from the children’s domicile (in green), and measurements where the substation is in the same building of the children’s domicile or adjacent to the children’s domicile (in red).

The last comparison analyzed in this paper was on the three groups divided by the distance between the children’s domicile and the ML/LV (20 kV/400 V) substation, as shown above in Table 2. The results of the estimated kernel density functions are presented in Figure 8 for each subgroup and considering day and night separately. The most significant results would be the ones regarding the night segments, when the children were actually at home and the distance from the substation is constant. It can be seen that, for the night, the kernel density function of the segments’ mean did not change significantly between the three groups. In particular, the mean’s curve of the segments’ level of magnetic field of the children whose domicile was in the same building or adjacent to the substation, was similar to the group where the substation is far from the children’s domicile. This could lead to the conclusion that the presence of the ML/LV (20 kV/400 V) substation located nearby the children’s domicile did not condition the level of the magnetic field. However, the *p*-value histograms obtained from the comparison among all the groups of the four parameters together for both daytime and night time showed a clustering of *p*-values near zero, so the three groups were statistically different. However, it should be noted that these subjects coming from the EXPERS study were selected randomly and not on the basis of distance from the ML/LV substation. Their relative position to electric networks was indeed determined by geo-localization after the random children’s selection. In the same manner, it must be highlighted that the numerosity of the three groups was not homogeneous (726 days for the group where the ML/LV substation is at least 40 m away from children’s domicile, 21 days for the group where the ML/LV substation is less than 40 m away from the children’s domicile, and 19 days for the group where the ML/LV substation is in the same building or adjacent to the children’s domicile, see also Table 2 above). In the future, it could be interesting to collect registrations based on the position of the ML/LV substation with respect to the children’s domicile in order to compare groups of similar size for confirming these results.

## 4. Conclusions

By the comparison between daily and nightly segments, the proposed stochastic modeling showed that the mean value (μ) of the level of magnetic field in the real exposure scenario of children was higher during the day than it was during the night. Moreover, the night segments showed a longer duration (*T*) and less variance (*σ*^2^) at higher values compared with the daily ones. This is explainable by the fact that, during the day, there is a presence of different activities with more variability, compared to the sleeping time. This resulted also in a statistically significant difference between the two groups, as shown by the shape of the *p*-value histogram. From these results, the segments were divided separately in daily and nightly for the other analysis as well.

The analysis regarding the age groups showed that, during both day and night, the three age groups were different among them, with children between 0 and 4 years old being more different in comparison with the other two groups of age, as can be seen from the KDE functions of the day and from the *p*-value histogram. This result could be explained because children at that age are less autonomous and more dependent on their parents compared with children aged 5 to 14, who have already started school. Our analysis showed that the age parameter is instead an important determinant of the children’s exposure, and it should therefore be used in future studies, where the children’s exposure should be analyzed considering the children’s age.

From the analysis on the groups based on the number of inhabitants, it can be seen that the groups living in towns with a low number of inhabitants presented characteristic more similar to the group of the rural area. For the daily segments, it can be seen that, up to about 10,000 inhabitants, there was no statistically significant difference among the groups. Similarly, cities with a number of inhabitants between 20,000 and 100,000 could be considered similar from the point of view of EMF exposure. This was truer for the daytime than for the night time. For the night time, in fact, the exposure differences remained for each class of inhabitants and for the rural area. Another important piece of information is that in bigger cities the level of exposure has been found higher with respect to smaller cities. This demonstrates that the level of urbanity is a relevant parameter that must be taken into consideration in the characterization of children’s exposure. This result is in line with the previous study of Magne and colleagues [24].

By means of the stochastic approach, the data recorded in Milan and Basel using the ARIMMORA protocol have been compared with similar data collected using the EXPERS protocol in France towns, to investigate the possible influence on the measurements due to differences in the measurements’ protocols. In particular, additional considerations come from the comparison of data coming from Milan and Basel with the corresponding groups of inhabitants in the EXPERS database. In the case of the city of Milan, although the level of exposition during the day was similar to the corresponding France group, the calculation of the *p*-value histograms indicated that it was not possible to characterize a general model for describing and summarizing the two groups together. The same conclusions were made regarding the comparison between Basel and the corresponding group of inhabitants in the EXPERS database. The level of magnetic field in Switzerland was indeed higher than the corresponding group in France. The reason for the higher level of magnetic field could be that, in the ARIMMORA measurement’s campaign in Basel, there was no random children selection but in fact an oversampling of potentially highly exposed children, selected because they lived close to power lines. The different time sampling between the two databases probably also caused the differences in the curves regarding the duration *T* parameters of the two groups. In this work, the EXPERS data were not down-sampled because this causes a loss of information regarding the other parameters. In the future, the possibility to have the same time sampling will help to make a more specific comparison.

The distance from the MV/LV (20 kV/400 V) substation seemed to influence the ELF-MF exposure, as the *p*-value histograms had a cluster of values near zero for all the three groups. This result is partially in agreement with the study of Magne et al. (2017) [24], where the 24 h mean magnetic field exposure was found to be correlated when the home was located less than 40 m away from an MV/LV substation in building, but not correlated if the substation is in the same building or adjacent.

In conclusion, this study demonstrates that stochastic modeling is a powerful tool for characterizing children’s exposure to low-frequency magnetic fields, and able to identify parameters that most affect the level of children’s exposure. The evaluation of the *p*-value histograms also indicates that characterizing a general model able to summarize and describe different subgroups is very improbable. The investigated features were indeed discriminant characteristics for determining children’s exposure. The proposed method can be used in the future for new data or for new subdivisions to identify new characteristic parameters.

However, it should not be forgotten, as was mentioned in the introduction, that the causal association between exposure to low-frequency magnetic fields and the possible increased risk of childhood leukemia has not been demonstrated [7]. For this reason, the parameters useful for the characterization of the exposure from the dosimetric point of view could be very different from those currently used to evaluate the exposure itself. For example, the climate and temperature could be two further parameters that could be important in the characterization of children’s exposure. Unfortunately, they were not originally included in the protocols of the two projects (EXPERS and ARIMMORA) in which the data were collected. However, they could easily be considered in the approach proposed in this study, and for this it is suggested they be considered in the design of future studies aimed at characterizing children’s exposure to magnetic fields.

## Figures and Tables

**Table 1:** ELF-MF exposure databases.

ARIMMORA Database		EXPERS Database	
Milan	Basel	Paris	France
Winter: 86 recordings	Winter: 79 recordings	29 recordings	948 recordings
Summer: 86 recordings	Summer: 80 recordings		
331 recordings from 166 children		977 recordings from 977 children	

**Table 2:** Subdivision of the two databases to conduct analysis of children’s ELF-MF exposure.

Type of Comparison	ARIMMORA Database		EXPERS Database	
Day vs. Night	682 days	Day: 4129 segments Night: 693 segments	767 days	Day: 38,742 segments
				Night: 8906 segments
Age’s Groups	0–4 years 0 days	No Registrations	0–4 years 175 days	Day: 7538 segments
				Night: 1901 segments
	5–9 years 405 days	Day: 2386 segments Night: 372 segments	5–9 years 244 days	Day: 12,381 segments
				Night: 3066 segments
	10–14 years 277 days	Day: 1743 segments	10–14 years 348 days	Day: 18,823 segments
		Night: 321 segments		Night: 3939 segments
Number of Inhabitants	Milan 366 days	Day: 2271 segments Night: 485 segments	Paris 28 days	Day: 1023 segments
				Night: 168 segments
			Rural Area 176 days	Day: 9868 segments
				Night: 2069 segments
			2000–4999 inhab. 121 days	Day: 6845 segments
				Night: 1579 segments
			5000–9999 inhab. 86 days	Day: 4987 segments
				Night: 822 segments
	Basel 316 days	Day: 1858 segments Night: 208 segments	10,000–19,999 inhab. 103 days	Day: 5776 segments
				Night: 1372 segments
			20,000–49,999 inhab. 90 days	Day: 3940 segments
				Night: 1158 segments
			50,000–99,999 inhab. 62 days	Day: 2617 segments
				Night: 671 segments
			100,000–199,999 inhab. 44 days	Day: 2052 segments
				Night: 509 segments
			200,000–1,999,999 inhab. 54 days	Day: 1634 segments
				Night: 558 segments
Distance from MV/LV (20 kV/400 V) substation	Not Available		>40 m 726 days	Day: 37,124 segments
				Night: 8277 segments
			<40 m 21 days	Day: 762 segments
				Night: 174 segments
			Same Building or Adjacent 19 days	Day: 842 segments
				Night: 263 segments
